# Sustainable co-production of 1,3-PDO, ethanol and H_2_ from glycerol via dark fermentation by *Citrobacter telavivensis* T1.2D-1 isolated from the deep subsurface

**DOI:** 10.3389/fbioe.2026.1778768

**Published:** 2026-04-20

**Authors:** Inés Ochoa-Arizu, Adrián Martínez-Bonilla, Nuria Rodríguez, Patricia Rojas, Ricardo Amils, José Luis Sanz, Alberto G. Fairén, David Ruano-Gallego

**Affiliations:** 1 Departamento de Biología Molecular, Universidad Autónoma de Madrid, Madrid, Spain; 2 IUBM-UAM and Centro de Biología Molecular Severo Ochoa (CSIC-UAM), Madrid, Spain; 3 Department of Planetology and Habitability, Centro de Astrobiología (CAB), CSIC-INTA, Madrid, Spain; 4 Department of Astronomy, Cornell University, Ithaca, NY, United States

**Keywords:** 1,3-propanediol, *Citrobacter*, deep subsurface, ethanol, hydrogen, Iberian Pyrite Belt

## Abstract

The increasing production of biodiesel has led to a surplus of glycerol, a polluting by-product in need of valorization. In this study, we demonstrate that *Citrobacter telavivensis* T1.2D-1, an extremophile bacterium isolated from the Iberian Pyrite Belt, effectively converts glycerol into valuable compounds via dark anaerobic fermentation. Genomic and bioinformatic analyses confirmed the presence of the *dha* and *pdu* operons, responsible for 1,3-propanediol (1,3-PDO) synthesis, and the *hyc* operon and *fdhF* gene involved in hydrogen (H_2_) production. Batch fermentations revealed that optimal yields of both H_2_ (0.94 mol^.^mol-glycerol^-1^) and 1,3-PDO (0.66 mol‧mol-glycerol^-1^) were achieved at 25 °C using 2 g L^-1^ of supplied glycerol. Optimum yield of ethanol (1 mol‧mol-glycerol^-1^) was achieved using 12.5 g L^-1^ of supplied glycerol. Interestingly, 1,3-PDO and H_2_ production inversely correlated with ethanol formation, suggesting metabolic competition. Antibiotic sensitivity profiling revealed susceptibility to multiple antibiotics, supporting future genetic engineering efforts. We suggest opperating with reactors at low concentrations to produce 1,3-PDO and H_2_ with high yields, and at medium concentrations to generate ethanol. Our findings support *C. telavivensis* T1.2D-1 as a promising venue for the sustainable biotechnological production of biohydrogen and bio-based 1,3-PDO from glycerol, offering a dual solution to both energy demands and industrial waste management.

## 1 Introduction

Growing concerns about climate change and the depletion of non-renewable resources have led to calls for a progressive reduction in dependence on oil as a source of fuels and materials, as outlined in the Paris Agreement (UNFCCC, 2015). Oil-derived chemical processes must be gradually replaced by biotechnological alternatives to achieve a circular bioeconomy and cleaner energy sources. Biodiesel, an increasingly important renewable fuel, is produced through the transesterification of vegetable oils and animal fats. Regrettably, for every ten volumes of biodiesel generated, one volume of glycerol is produced as waste (Tan, et al., 2013; Zhang, et al., 2016). If released into the environment, glycerol can contaminate soils and water sources and contribute to emissions of a greenhouse gas, methane (Dzulkarnain, et al., 2022). Another renewable fuel, biohydrogen, is expected to be used in industries such as iron and steel manufacturing, as well as in transportation. Hydrogen contains three times more energy (142 MJ kg^-1^) than gasoline (48 MJ kg^-1^), and its combustion produces only water as a by-product (Dulta, et al., 2022). Currently, hydrogen accounts for 1% of the U.S. energy consumption; however, most of this hydrogen is produced from fossil fuels that release CO_2_, and the deployment of other cleaner H_2_ technologies is still in the very early stages (Victor and Nichols, 2024).

A renewable method for producing hydrogen (H_2_) is anaerobic dark fermentation using glycerol, thereby valorizing a waste product derived from biodiesel production. In this process, microorganisms utilize the hydrogen-lyase complex (FHL complex), which consists of seven subunits that oxidize formic acid, reducing protons to generate hydrogen and carbon dioxide (McDowall et al., 2014). Dark fermentation offers certain advantages for H_2_ production compared to other methods, such as direct photolysis. Notably, it does not require a light source; however, its efficiency is relatively low (Chen et al., 2023). Nevertheless, dark fermentation is not inhibited by the presence of hydrogen and enables the valorization of waste products. Potential substrates for use in bioreactors include lignocellulosic feedstocks or other carbohydrate-rich materials.

**FIGURE 1:**
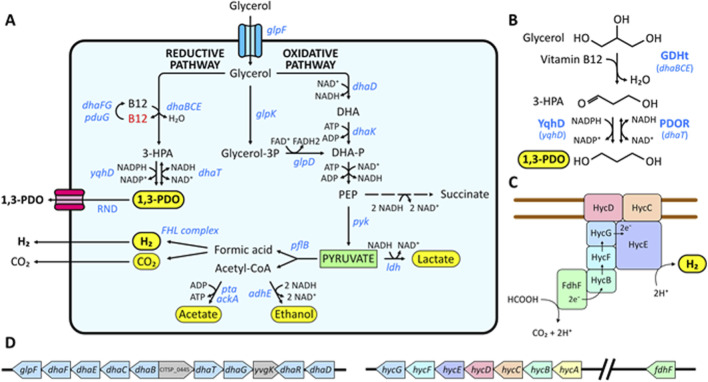
**(A)** Inferred pathway of glycerol in *C. telavivensis* T1.2D-1. Glycerol enters through a membrane transporter and can follow two routes, the reductive or the oxidative. **(B)** Conversion of glycerol to 1,3-PDO. In the first step, glycerol dehydratase (*dhaB*, *dhaC* and *dhaE)* catalyzes the production of 3-hydroxypropionaldehyde (3-HPA). In a second step, 1,3-PDO dehydrogenase (*dhaT)* converts 3-HPA to 1,3-PDO reversibly. The alternative dehydrogenase encoded in *yqhD* performs this reaction irreversibly. **(C)** Schematic representation of the FHL complex. **(D)** Schematic representation of the *dha* and *hyc* operons in the genome of *C. telavivensis* T1.2D-1. Arrow at one end: non-reversible reaction. Double arrow: reversible reaction. End products are highlighted in yellow and genes indicated in blue.

When used for hydrogen (H_2_) production, glycerol generates 1 mol of NADH upon oxidation to glyceraldehyde-3-phosphate (glyceraldehyde-3-P). To achieve respiratory balance, some bacteria regenerate NAD^+^ by reducing glyceraldehyde-3-P to 1,3-propanediol (1,3-PDO) (Tey et al., 2023). 1,3-PDO is used in the chemical industry (e.g., production of solvents, adhesives, inks, lubricants, cosmetics, antifreezes, etc.) as well as in the textile industry, where it serves as a monomer for the synthesis of polytrimethylene terephthalate (PTT) (Saltzberg et al., 2016). The global market for 1,3-PDO was valued at $424.8 million in 2023 (Gran View Research, 2023). Currently, DuPont produces 1,3-PDO using *E. coli* that has been genetically engineered with genes from *Klebsiella pneumoniae* encoded in the *dha* operon. The DhaBCE complex first converts glycerol into 3-hydroxypropanal (3-HPA), an intermediate that is subsequently converted to 1,3-PDO by the enzyme DhaT (Figure 1). The DhaBCE complex is then reactivated by DhaFG. This biotechnological route is progressively replacing petroleum-derived sources, thereby reducing greenhouse gas emissions. However, it still relies on glucose as a substrate and employs a suboptimal host for gene expression. An alternative route, encoded in the *pdu* operon of certain *Citrobacter* and *Klebsiella* strains (Ainala et al., 2013), produces 3-HPA from glycerol as a secondary product (Tang et al., 2009). In this pathway, the PduCDE complex generates 3-HPA, and PduG is responsible for reactivating the complex. In *K. pneumoniae*, PduG can also reactivate DhaBCE in addition to DhaFG (Seo et al., 2009). Finally, *E. coli* and *K. pneumoniae* produce an alcohol dehydrogenase which can substitute for the activity of DhaT, encoded in locus *yqhD* (Celińska, 2010; da Silva et al., 2015).

Another product of glycerol fermentation is ethanol, which is also used as a low-energy biofuel blended with conventional fuels. The production of bioethanol from glycerol is a more cost-effective option, with reported yields ranging from 7 to 30 g L^-1^ (Elsayed et al., 2023). Furthermore, fed-batch cultivation has been shown to double the yield achieved by the batch process (Sunarno et al., 2020).

We recently reported the production of H_2_ by *Citrobacter telavivensis* T1.2D-1 during anaerobic growth on glycerol as the sole carbon source (Gallego-Rodríguez et al., 2023). *C telavivensis* T1.2D-1 was isolated from the deep subsurface of the Iberian Pyrite Belt (IPB) at a depth of 63.55 m. The Earth’s subsurface is considered an extreme environment due to the absence of light and oxygen, as well as its extreme oligotrophic conditions, and the microorganisms that inhabit this environment form what is known as the “dark biosphere.” These microorganisms possess unique characteristics that enable them to adapt to such extreme conditions, making them promising candidates for various biotechnological applications, including space exploration. The aim of this work was to evaluate the metabolic options of *C. telavivensis* T1.2D-1 through its genomic analysis identifying the genomic loci responsible for glycerol fermentation, assessing the efficiency of H_2_, 1,3-PDO and ethanol production at different glycerol concentrations and temperatures, and to identify its antibiotic sensitivity for future genetic engineering experiments to improve the efficiency in the production of the identified products of biotechnological interest.

## 2 Materials and methods

### 2.1 Genomic analysis of *Citrobacter telavivensis* T1.2D-1

Both the genomic information and the annotation of *Citrobacter telavivensis* T1.2D-1 and *Citrobacter rodentium* ICC168 can be found in the National Center for Biotechnology Information (NCBI) with the accession numbers PRJEB60615 and PRJEA34685, respectively. The genes related to 1,3-PDO and H_2_ production in *Citrobacter freundii* and *Escherichia coli* K12 and their respective amino acid sequences were obtained in European Nucleotide Archive (ENA) and Uniprot using the accession numbers specified in Table 1. The genes were aligned against the genomes using nucleotide BLAST and the proteins against their annotations using protein BLAST, in both cases with the default parameters. In the first case, we considered as positive those results with a coverage greater than 85%, a sequence identity greater than 60% and an E value lower than 1e-10. In the case of the amino acids, we considered positive hits those with a coverage greater than 85%, a sequence identity greater than 60% and an E-value lower than 1e-10.

**TABLE 1:** Genes identified in this study in the genome of *C. telavivensis* T1.2D-1 related to 1,3-PDO and H_2_ production.

Gene	Codified enzyme	Accession	*C. telavivensis T1.2D-1*	Function
*dhaB*	Glycerol dehydratase large subunit	Uniprot: P45514 ENA: SYX43571	CAI9393044.1	Transforms glycerol into 3-hydroxypropanal. Vitamin B12 as a cofactor
*dhaC*	Glycerol dehydratase middle subunit	Uniprot: A8CH91 ENA: ABA39277.1	CAI9393042.1	
*dhaE*	Glycerol dehydratase small subunit	Uniprot: A8CH95 ENA: AAB48852.1	CAI9393039.1	
*dhaF*	Glycerol dehydratase reactivation factor large subunit	Uniprot: Q46035 ENA: ABI36568	CAI9393036.1	Reactivates inhibited glycerol dehydratase
*dhaG*	Glycerol dehydratase small subunit	Uniprot: P45516 ENA: ABI36569	CAI9393053.1	
*dhaT*	1,3-Propanediol dehydrogenase	Uniprot: P45513 ENA: HBC6240007	CAI9393050.1	Reduces 3-hydroxypropanal to 1,3-propanediol. Reversible enzyme
*dhaD*	Glycerol dehydrogenase	Uniprot: P45511 ENA: SYX25694	CAI9393062.1	Catalyzes NAD-dependent oxidation of glycerol to dihydroxyacetone
*dhaK*	Dihydroxyacetone kinase	Uniprot: P45510 ENA: DQ473522	CAI9390316.1	Catalyzes dihydroxyacetone phosphorylation
*pduC*	Propanediol dehydratase large subunit	Uniprot: P0DUM7 ENA: HBC6113957	CAI9396235.1	Catalyzes the dehydration of 1,2-propanediol into propionaldehyde. Requires vitamin B12 as a cofactor
*pduD*	Propanediol dehydratase middle subunit	Uniprot: P0DUM8 ENA: HBC8116818	CAI9396237.1	
*pduE*	Propanediol dehydratase small subunit	Uniprot: P0DUM9 ENA: HBC7084927	CAI9396239.1	
*pduG*	Propanediol dehydratase reactivator large subunit	Uniprot: B1VB67 ENA: GAL39426	CAI9396241.1	Reactivates the inhibited propanediol dehydratase enzyme
*yqhD*	Alcohol dehydrogenase	Uniprot: Q46856 ENA: NUV03863	CAI9392804.1	Catalyzes aldehyde reduction
*fdhF*	Formate dehydrogenase H	Uniprot: P07658 ENA: M13563	CAI9390481.1	Breaks down formic acid to H_2_ and CO_2_ under anaerobic conditions in the absence of exogenous electron acceptors
*hycC*	Formate hydrogenolyase unit 3	Uniprot: P16429 ENA: QGJ09820	CAI9387359.1	Integral membrane proteins that anchor the FHL complex on the cytoplasmic side of the membrane
*hycD*	Formate hydrogenolyase unit 4	Uniprot: P16430 ENA: WNS15254	CAI9387356.1	
*hycB*	Formate hydrogenolyase unit 2	Uniprot: P0AAK1 ENA: WNS17627	CAI9387362.1	Electron transfer in the FHL complex
*hycF*	Formate hydrogenolyase unit 6	Uniprot: P16432 ENA: WNR85486	CAI9387350.1	
*hycG*	Formate hydrogenolyase unit 7	Uniprot: P16433 ENA: WNS15251	CAI9387347.1	
*hycE*	Formate hydrogenolyase unit 5	Uniprot: P16431 ENA: WNR91071	CAI9387353.1	Reduction of protons to H_2_

### 2.2 Growth conditions of *C. telavivensis* T1.2D-1

*C. telavivensis* T1.2D-1 was grown in aerobic conditions using Tryptic Soy Broth (TSB) (Condalab) and Luria Bertani (LB) (Sigma) broth at 37 °C and agitation of 170 rpm. Anaerobic media was prepared by boiling distilled water, dissolving the salts in this water and cooling it down under a N_2_ atmosphere inside a serum bottle. The serum bottles were sealed using a butyl rubber stopper and crimped with aluminum caps. All media were autoclaved at 121 °C for 15 min. LB solid medium was also prepared in aerobic conditions and poured into Petri dishes right after the sterilization.

For medium M9 preparation, three different solutions were prepared: MgSO_4_ (1M), CaCl_2_ (0.1 M) and M9 salts (containing 60 g of Na_2_HPO_4_, 30 g of KH_2_PO_4_, 10 g of NH_4_Cl and 5 g of NaCl). All compounds from Sigma. These solutions were prepared in anaerobic conditions separately and autoclaved. Additionally, a glycerol (Sigma) solution was prepared in anaerobic conditions and filter-sterilized using a 0.22 μm pore size syringe filter (Millex). After autoclaving, the solutions were mixed inside the glovebox under a N_2_ atmosphere using the following ratio: 1 mL of MgSO_4_, 1 mL of CaCl_2_, 100 mL of M9 salts, the volume of the glycerol solution necessary to reach the desired final concentration and anaerobic distilled water up to 1 L and the pH adjusted to 7. Once the M9 medium had been prepared, 15 mL of the medium were poured into 30 mL sterile serum bottles and closed, as above.

A total of 54 bottles were filled with anaerobic minimal M9 medium supplemented with glycerol as the only carbon source at three concentrations (2 g L^-1^, 25 g L^-1^, and 60 g L^-1^). After inoculation these bottles were incubated at 25 °C, 30 °C, or 37 °C for up to 22 days. For each set of conditions, in duplicate, one bottle was used to monitor optical density (OD), one to measure H_2_ concentration, and one to determine the concentrations of 1,3-PDO and glycerol. At different times 1 mL was extracted from the correspondent bottle to measure OD and 1 mL to measure 1,3-PDO and glycerol. This approach minimized alterations to the microenvironment during sampling. For the fed-batch cultures 10 bottles of anaerobic M9 medium were supplemented with 3, 5, 11.5, 12.5 and 16 g L^-1^ of glycerol, in duplicate, inoculated and incubated at 25 °C. Glycerol consumption, as well as the production of 1,3-PDO and ethanol, were measured every 24 h over a period of 5 days. To inoculate the serum bottles, cultures grown in aerobic TSB medium were transferred into anaerobic M9 medium and grown for 24 h at 25 °C at 130 rpm in a shaker. To ensure anaerobic conditions, a second subculture was incubated before dilution to Optical Density (O.D.) at 600 nm of 0.05. The anaerobic media were kept static and at the corresponding temperature for each study. At the end of the experiments, the pH of the medium was measured with the pH meter (Thermo Orion 920A Plus Advanced ISE/PH/MV/ORP Meter). All the experiments were performed in duplicate.

### 2.3 Analytical methods

1 mL samples were centrifuged at 6,000 *g* for 5 min and the supernatant was used for the measurement of glycerol and fermentation by-products, 1,3-PDO and ethanol, using a HPLC 1200 Infinity Series (Agilent technologies), with a refractive index detector and a MetaCarb 67H column (Agilent Technologies). The flow rate of the mobile phase (0.01 N H_2_SO_4_) was 0.65 mL min^-1^ The resolution for both polyols was high, 1 ppm for glycerol and 10 ppm for 1,3-PDO.

Gas composition of the serum bottles’ headspace was determined using a Bruker Series Bypass 450-GC gas chromatograph equipped with a column CP2056 0.6 m x 1/8″ Ultimetal Cromsorb GPH 100–120 mesh, and a TCD (H_2_ and CO_2_) and a FID (CH_4_) detectors. N_2_ was used as carrier gas. The GC was calibrated using a Luxfer gas cylinder 950-090445 (CALGAZ Ltd, United Kingdom) containing Methane 40%, Hydrogen 40%, and Carbon dioxide balance.

### 2.4 Identification of antibiotic resistance

To determine the sensitivity of *C. telavivensis* T1.2D-1, an antibiogram analysis was conducted. LB-agar plates were inoculated with 200 μL of a culture grown overnight in aerobic LB broth of C*. telavivensis* T1.2D-1, which were then spread using disposable L-shaped bacteriology loops (VWR). Then, discs containing the following antibiotics were placed on the inoculated Petri dishes: nalidixic acid (30 μg/disc), streptomycin (10, 32, 64, 128, 256 and 500 μg/disc), trimethoprim (5 μg/disc), cephalotin (30 μg/disc), erythromycin (15 μg/disc), tetracycline (30 μg/disc), chloramphenicol (30 μg/disc), gentamicin (10 μg/disc) and kanamycin (50 μg/disc). The antibiotic discs were purchased from BBl. After overnight incubation at 37 °C, the diameter of the halo formed by each antibiotic was measured. If greater than 15 mm, the strain was considered sensitive for that antibiotic.

The analysis of antibiograms was also conducted in liquid medium using different concentrations of the same antibiotic. *C. telavivensis* T1.2D-1 was first grown overnight in liquid TSB medium as previously described. Then it was transferred into 96-well plates at a final O.D. at 600 nm of 0.05 using fresh TSB medium. The wells were supplemented with different dilutions (0.5, 1, 2, 4, 8, 16, 32, 64, 128, 256 and 512 μg/mL) of kanamycin, streptomycin, ampicillin, tetracyclin, gentamycin and chloramphenicol. Antibiotics were purchased from Sigma. Each concentration was prepared in duplicates. A negative control (without bacteria) and a positive control (without antibiotics) were also prepared in duplicates. The plates were incubated at 37 °C and the O.D. was measured using a CLARIOstar Plus microplate reader (BMG Labtech).

## 3 Results

### 3.1 Identification of genes involved in the generation of 1,3-PDO and H_2_

The annotated genome of *C. telavivensis* T1.2D-1 consists of a single bacterial chromosome with a length of 5,435,711 bp and a GC content of 53.56%. It contains 5,084 coding sequences, of which 1,264 are hypothetical proteins (Gallego-Rodríguez et al., 2023). Based on our preliminary data on the production of H_2_ by *C. telavivensis* T1.2D-1, we sought to identify the genomic loci responsible for glycerol fermentation and those associated with the reduction of glyceraldehyde-3-phosphate. To this end, we aligned the genes and proteins responsible for H_2_ and 1,3-propanediol (1,3-PDO) generation in producing strains, using *E. coli* K12 and *Citrobacter freundii* as templates. Our analysis confirmed that the strain encodes all genes required for H_2_ production, in addition to the *dha* and *pdu* operons, which are responsible for the generation of 1,3-PDO (Figure 1; see Table 1 for details).

To identify the essential genes required for growth and for the production of H_2_ and 1,3-PDO from glycerol as the sole substrate, a genomic analysis identical to that performed for *C. telavivensis* T1.2D-1 was conducted on *Citrobacter rodentium* ICC168-a bacterium of the same genus that is unable to grow on glycerol under anaerobic conditions. All genes present in *C. telavivensis* T1.2D-1 were identified in *C. rodentium* ICC168 except for the *dha* genes (Supplementary Table S1), indicating that these genes are essential for the growth of *C. telavivensis* T1.2D-1 on glycerol.

### 3.2 Optimization of H_2_ and 1,3-PDO production in C. telavivensis T1.2D-1

**FIGURE 2:**
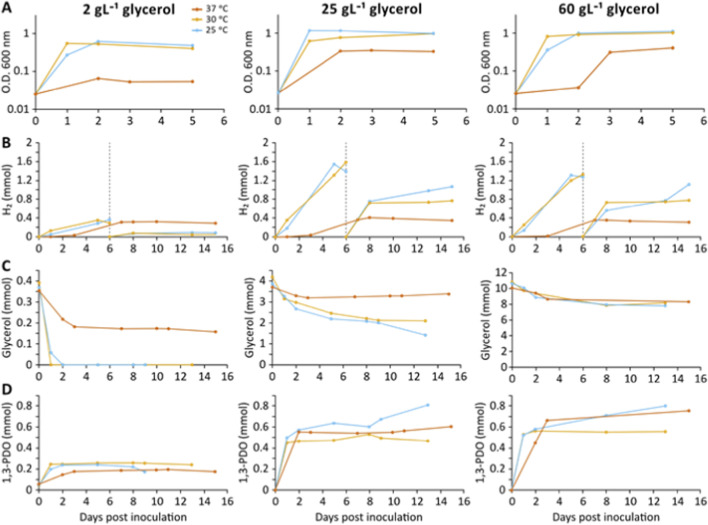
**(A)** Anaerobic growth, **(B)** H_2_ production, **(C)** Glycerol consumption and **(D)** 1,3-PDO production of *C. telavivensis* T1.2D-1. These parameters were measured at initial glycerol concentrations of 2, 25 and 60 g·L^-1^ and at temperatures of 25, 30 and 37 °C. Experiments in duplicate, average values represented.

We began by determining whether the production of 1,3-PDO was associated with the observed generation of H_2_ under anaerobic conditions by *C. telavivensis* T1.2D-1 at different glycerol concentrations and temperatures (Figure 2). At 25 °C it took 2 days to reach the stationary phase when using an initial concentration of 2 and 25 g L^-1^ glycerol, and only 1 day with 60 g L^-1^. At 30 °C the stationary phase was achieved with only 1 day, regardless the initial glycerol concentration. Growth at 37 °C was limited when using an initial concentration of 2 g L^-1^ of glycerol, while the stationary phase was reached in 3 days with 25 g L^-1^ and in 2 days with 60 g L^-1^ initial concentrations of glycerol (Figure 2A). Regarding H_2_ measurements (Figure 2B), we observed that at 37 °C, production ceased on day six at 0.3 mmol, regardless of the initial glycerol concentration. At 25 °C and 30 °C, H_2_ production was similar and considerably higher at glycerol concentrations of 25 and 60 g L^-1^. On day six, bottles incubated at 25 °C and 30 °C had stabilized, so the headspace was re-gassed, restoring the original N_2_ atmosphere to evaluate the evolution of H_2_ production. The rate of H_2_ production was maximal until day eight at both temperatures; although production continued at a slower rate thereafter at 25 °C (Figure 2B).

We also observed that hydrogen (H_2_) production was associated with glycerol depletion (Figure 2C) and 1,3-propanediol (1,3-PDO) formation (Figure 2D). At 30 °C, 1,3-PDO generation ceased after 24 h, reaching 0.25 mmol when the initial glycerol concentration was 2 g L^-1^. A similar cessation was observed at 25 °C on the second day and at 37 °C on the third day, with the maximum 1,3-PDO concentration reaching 0.2 mmol in the latter case. However, when the initial glycerol concentration was increased to 25 g L^-1^, 1,3-PDO production exceeded 0.5 mmol within 24 h at both 25 °C and 30 °C. Subsequently, at 25 °C, 1,3-PDO production continued, albeit at a lower rate. A similar trend was observed at 25^o^C and 30^o^C with an initial glycerol concentration of 60 g L^-1^, curiously reaching a comparable peak. In contrast, at 37 °C and 25 g L^-1^ glycerol, the production peak of 0.55 mmol was reached after 2 days of incubation.

With respect to glycerol measurements (Figure 2C), we observed that at 30 °C and 25 °C, the initial 2 g L^-1^ of glycerol was consumed within one and 2 days, respectively. At 37 °C, glycerol consumption ceased after 3 days, regardless of the initial concentration. In the bottles containing 25 g L^-1^ and 60 g·L^-1^ glycerol at 25 °C and 30 °C, we observed maximum glycerol consumption by day one and day two, respectively. However, glycerol consumption continued at a slower rate in the subsequent days.

### 3.3 Higher 1,3-PDO production is associated with lower fermentation via ethanol

**FIGURE 3:**
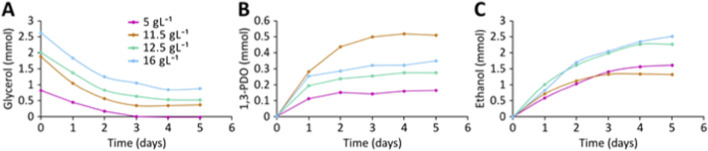
**(A)** Glycerol consumption, **(B)** production of 1,3-PDO and **(C)** ethanol production of *C. telavivensis* T1.2D-1 at the indicated initial concentrations of glycerol. Experiments in duplicate, average values represented.

As we observed continuous glycerol consumption-even in situations where the production of H_2_ and 1,3-PDO decreased or ceased (Figure 2D), we hypothesized that other metabolic products derived from glycerol might be produced. Since ethanol is an alternative fermentation product to H_2_ in *C. telavivensis* T1.2D-1, as inferred from its genome analysis (Figure 1), it could also be a byproduct of anaerobic growth. Furthermore, we aimed to determine the optimal initial concentration of glycerol, between 2 and 25 g L^-1^. To this end, ten bottles of anaerobic M9 medium were supplemented with 3, 5, 11.5, 12.5, and 16 g L^-1^ of glycerol, in duplicates, inoculated, and incubated at 25 °C. Glycerol consumption, as well as the production of 1,3-PDO and ethanol, were measured every 24 h over a period of 5 days (Figure 3). In cultures with an initial glycerol concentration of 5 g L^-1^, no residual glycerol was detected by the end of the experiment. When the initial substrate concentration was 11.5 g L^-1^ or higher, glycerol was not completely exhausted; its consumption ceased after day 3 for the 11.5 g L^-1^ cultures and after day 4 for those with 12.5 and 16 g L^-1^ (Figure 3A).

Production of 1,3-PDO reached a maximum when 11.5 g L^-1^ of initial glycerol was used, attaining 0.5 mmol by day 3 (Figure 3B). After this point, production ceased, mirroring the pattern observed for glycerol consumption. For all other initial glycerol concentrations, 1,3-PDO was produced at high rates up to day 1, after which production either decreased or, in the case of 5 g L^-1^, stopped entirely. Interestingly, ethanol production was observed and was inversely correlated with 1,3-PDO production (Figure 3C). When 12.5 g L^-1^ of initial glycerol was used, 1,3-PDO dropped to 0.27 mmol, approximately half the production at 11.5 g L^-1^ glycerol. At this glycerol concentration ethanol production reached 1 molmol-1 glycine.

### 3.4 C. telavivensis T1.2D-1 is sensitive to common antibiotics

Given these results, *C. telavivensis* T1.2D-1 appears to be a promising candidate for the optimized production of 1,3-PDO, ethanol, or H_2_ through transformation and metabolic engineering. To assess its sensitivity to a range of common antibiotics, we performed an antibiogram by seeding agar plates with a lawn of the strains and placing standardized, pretreated antibiotic discs onto the surface, as shown in Supplementary Table S2. The antibiotic resistance measured using antibiotic discs showed an interesting display of sensitivities, being nalidixic acid, trimethoprim, tetracycline and chloramphenicol the antibiotics that require higher concentrations to inhibit cell growth. A more detailed analysis was conducted based on resistance genes present in commonly used laboratory plasmids and their associated antibiotic resistance profiles. This assay was performed in liquid medium across a range of antibiotic concentrations, with endpoint optical density (O.D.) measurements recorded (Supplementary Table S3). As can be seen *C. telavivensis* is very sensitive in liquid medium to low concentration of tetrqacycline, chloramphenicol and gentamycin, while extremely resistant to ampicillin.

## 4 Discussion

*C. telavivensis* T1.2D-1 was isolated from the subsurface of the Iberian Pyrite Belt (Gallego-Rodríguez et al., 2023) and demonstrates promising potential for biotechnological applications. This proteobacterium is capable of growth on minimal carbon sources across a range of temperatures and under anaerobic conditions. As a facultative anaerobe, *C. telavivensis* T1.2D-1 possesses a competitive advantage over strictly anaerobic H_2_-producing strains, such as *Clostridium* (Yun et al., 2022). In industrial settings, facultative anaerobes are particularly attractive because they can be cultivated rapidly under aerobic conditions to achieve high cell densities, and subsequently shifted to anaerobic conditions for the production of desired fermentation products once the oxygen supply is halted. Glycerol, a byproduct of biodiesel production, can be revalorized as a substrate for the growth of biotechnologically relevant microorganisms, thereby reducing its environmental impact. Additionally, the adaptability of *C. telavivensis* T1.2D-1 allows it to maintain activity even when oxygen infiltrates bioreactors. In this study, we show that *C. telavivensis* T1.2D-1 grows and produces H_2_, ethanol, and 1,3-propanediol optimally under anaerobic conditions at 25 °C, requiring minimal substrates (Figure 2). This optimal temperature is close to that of the subsurface from which it was isolated (Gallego-Rodríguez et al., 2023). This characteristic offers a significant advantage in industrial contexts by reducing both the energy consumption and the operational costs associated with bioreactors.

Our data indicate that operating at lower glycerol concentrations yields the highest productivities and maximizes glycerol utilization. At 2 g glycerol·L^-1^, glycerol consumption is complete, and the yields of 1,3-PDO and H_2_ are 0.66 mol 1,3-PDO·mol^-1^ glycerol and 0.94 mol H_2_·mol^-1^ glycerol, respectively. These yields surpass those reported for many Enterobacteriaceae strains (Yang et al., 2017). However, at 25 g glycerol·L^-1^, only 50% of the glycerol is consumed, and the yields decrease to 0.14 mol 1,3-PDO and 0.6 mol H_2_ per mole of glycerol supplied. At 60 g glycerol·L^-1^, although there appears to be no inhibition of *C. telavivensis* growth (Figure 2), only one third of the glycerol is consumed, resulting in absolute quantities of 1,3-PDO and H_2_ similar to those obtained at 25 g glycerol·L^-1^. Additionally, the strain tolerates high glycerol concentrations up to 127 g L^-1^ (data not shown), albeit with reduced growth, indicating resilience and flexibility for industrial-scale processes.

The yields achieved using low concentrations of glycerol are higher than those reported in the literature for hydrogen, which range between 0.50 and 0.67 mol H_2_ per mol of glucose (Lo et al., 2013; Kumar et al., 2015). Additionally, the yields obtained are only slightly lower than 0.72 mol of 1,3-PDO per mol of glycerol consumed, which is the theoretical maximum yield produced by bacterial fermentation of glycerol (Zeng, 1996). Therefore, in this work, we propose the production of 1,3-PDO and H_2_ using continuously operated bioreactors with low glycerol concentrations, while optimizing hydraulic retention times to the lowest possible values.

Regarding ethanol production, the maximum yield (1 mol per mol of glycerol supplied) was obtained at an initial concentration of 12.5 g L^-1^ (Figure 3), which is higher than yields reported by other authors (Ito et al., 2005). These findings further support the potential of *C. telavivensis* T1.2D-1 for the industrial production of value-added products. As a proof of concept, we simulated a discontinuous fed-batch system, supplying a daily glycerol concentration of 2 g L^-1^ for 5 days. These preliminary results suggest that the yields of 1,3-PDO and ethanol could be further increased by minimizing substrate or product inhibition.

Given that these results are based on the use of a single strain, bioprocesses can be better controlled in bioreactors when compared to those employing undefined microbial communities (Cordeiro et al., 2023). Given the industrial potential of *C. telavivensis* T1.2D-1, we evaluated the antibiotic sensitivity of this strain to common antibiotics (Supplementary Tables S2,S3) typically used in plasmids for genetic engineering. Genomic analysis revealed that, in addition to the *dha* operon (Figure 1A), this strain encodes *yqhD*, an alcohol dehydrogenase that can substitute for DhaT in the production of 1,3-PDO in Enterobacteriaceae. Expression of YqhD is preferred for optimizing 1,3-PDO yield in *E. coli* compared to identical strains utilizing *dhaT* (Tan et al., 2013; He et al.,. 2017). For example, overexpression of *E. coli ydhD* in *K. pneumoniae* increased 1,3-PDO production by 125% (Seo et al., 2009). Unlike DhaT, YqhD utilizes both NADH and NADPH, rather than only NADH, functions under both aerobic and anaerobic conditions, and catalyzes the irreversible conversion of 3-HPA to 1,3-PDO. These factors may contribute to achieving higher titers of 1,3-PDO in *C. telavivensis* T1.2D-1. Alternatively, given the high ethanol titers obtained with *C. telavivensis* T1.2D-1, *adhE* could be overexpressed while deleting the *dha* operon, thereby redirecting metabolism toward ethanol production.

## 5 Conclusion

This study highlights the biotechnological potential of *Citrobacter telavivensis* T1.2D-1 as an efficient microbial platform for the co-production of 1,3-propanediol (1,3-PDO) and hydrogen (H_2_) from glycerol under anaerobic conditions. Through a comprehensive genomic and metabolic analysis, we confirmed the presence and functionality of key operons—*dha*, *pdu*, and *hyc*—involved in glycerol fermentation and energy generation. Notably, the strain encodes the *yqhD* gene, whose product offers an alternative and potentially more efficient route for 1,3-PDO biosynthesis, further supporting its industrial relevance.

Our optimization experiments demonstrated that 25 °C is the most favorable temperature for simultaneous production of 1,3-PDO, ethanol, and H_2_, 2 g L^-1^ of glycerol was the optimal substrate concentration to produce 1,3-PDO and H2 per mol of glycerol supplied, yielding 0.66 mol 1,3-PDO and 0.94 mol H2, values that are competitive or superior to many other Enterobacteriaceae. In the case of ethanol, the optimal concentration was 12.5 g^.^L´1 of glycerol, reaching 1 mol ethanol per mol of glycerol supplied.

Furthermore, we observed a clear inverse relationship between ethanol and 1,3-PDO production, suggesting potential metabolic trade-offs that can be redirected through genetic engineering. Suppressing ethanol production could enhance 1,3-PDO and H_2_ yields. The successful growth of the strain at various glycerol concentrations, and its response under simulated fed-batch conditions, paves the way for future process scale-up using continuous or semi-continuous fermentation systems.

It is worthy of note that the antibiotic sensitivity profile of *C. telavivensis* T1.2D-1 supports its amenability to genetic manipulation, facilitating the development of engineered strains with enhanced metabolic capacities. The creation of a Δ*dhaT* mutant and the identification of common resistance markers provide a foundation for future metabolic engineering strategies targeting increased product selectivity and yield.

In conclusion, *C. telavivensis* T1.2D-1 is a promising microbial producer for sustainable 1,3-PDO, ethanol and H_2_ from glycerol, a biodiesel-derived waste. Its metabolic versatility, resilience to high substrate loads, and potential for genetic optimization make it as a strong candidate for deployment in industrial biotechnology. Our results suggest optimal performance when operating with reactors at low concentrations to produce 1,3-PDO and H_2_, and at medium concentrations to generate ethanol. Future studies should focus on metabolic modeling, pathway optimization, and bioreactor design to further enhance the strain’s performance and economic viability.
