# Supplementary material for: Sustainable co-production of 1,3-PDO, ethanol and H2 from glycerol via dark fermentation by Citrobacter telavivensis T1.2D-1 isolated from the deep subsurface
Source: Front Bioeng Biotechnol. 2026 Apr 20;14:1778768. doi: 10.3389/fbioe.2026.1778768 (PMC13136257; doi:10.3389/fbioe.2026.1778768)
Supplement: Supplementary file 1 [file Table1.docx]

Supplementary Material

# Supplementary Tables

# Table S1. Comparison of genes in *C. rodentium* ICC168 (Cr) and *C. telavivensis* T1.2D-1 (Ct)

|  | ***glpF*** | ***dhaB*** | ***dhaC*** | ***dhaE*** | ***dhaG*** | ***dhaF*** | ***dhaT*** | ***pduC*** | ***pduD*** | ***pduE*** | ***pduG*** | ***yqhD*** | ***RND*** | ***dhaD*** | ***dhaK*** | ***glpK*** | ***hycB*** | ***hycC*** | ***hycD*** | ***hycE*** | ***hycF*** | ***hycG*** | ***FdhF*** |  |
| --- | --- | --- | --- | --- | --- | --- | --- | --- | --- | --- | --- | --- | --- | --- | --- | --- | --- | --- | --- | --- | --- | --- | --- | --- |
| ***Cr*** | | ✓ | X | X | X | X | X | X | ✓ | ✓ | ✓ | ✓ | ✓ | ✓ | X | X | ✓ | ✓ | ✓ | ✓ | ✓ | ✓ | ✓ | ✓ |
| ***Ct*** | | ✓ | ✓ | ✓ | ✓ | ✓ | ✓ | ✓ | ✓ | ✓ | ✓ | ✓ | ✓ | ✓ | ✓ | ✓ | ✓ | ✓ | ✓ | ✓ | ✓ | ✓ | ✓ | ✓ |

# Table S2. Disk antibiotic sensitivity of *C. telavivensis* T1.2D-1.

| **Antibiotic** | **Dose per disc** | ***C. telavivensis*** | **Inhibitory effect** |
| --- | --- | --- | --- |
| **Nalidixic acid** | 30 µg | sensible | Replication (gyrase) |
| **Streptomycin** | 10 µg  32 µg  64 µg  128 µg  256 µg  500 µg | resistant  resistant  intermediate  sensible  sensible  sensible | Protein synthesis (30S) |
| **Trimethoprim** | 5 µg | sensible | Synthesis of folic acid |
| **Cefalotin** | 30 µg | resistant | Synthesis of peptidoglycan |
| **Erythromycin** | 15 µg | sensible | Protein synthesis (50S, peptidyl transferase) |
| **Tetracycline** | 30 µg | sensible | Protein synthesis (30S, tRNA attachment) |
| **Chloramphenicol** | 30 µg | sensible | Protein synthesis (50S, peptidyl transferase) |
| **Gentamycin** | 10 µg | resistant | Protein synthesis (30 S) |
| **Kanamycin** | 50 µg | resistant | Protein synthesis (30 S) |

# Table S3. Sensitivity to indicated antibiotics of *C. telavivensis* T1.2D-1 in liquid LB medium.

| **Antibiotic concentration (µg/mL)** | **512** | **256** | **128** | **64** | **32** | **16** | **8** | **4** | **2** | **1** | **0.5** |
| --- | --- | --- | --- | --- | --- | --- | --- | --- | --- | --- | --- |
| Kanamycin | S | S | S | S | R | R | R | R | R | R | R |
| Streptomycin | S | S | S | S | R | R | R | R | R | R | R |
| Ampicillin | R | R | R | R | R | R | R | R | R | R | R |
| Tetracycline | S | S | S | S | S | S | S | S | S | S | S |
| Gentamycin | S | S | S | S | S | S | S | R | R | R | R |
| Chloramphenicol | S | S | S | S | S | S | S | S | S | R | R |
